# Supplementary material for: Improving the diagnostic recognition of thoracic endometriosis: Spotlight on a new histo-morphological indicator
Source: PLoS One. 2021 May 13;16(5):e0251385. doi: 10.1371/journal.pone.0251385 (PMC8118331; doi:10.1371/journal.pone.0251385)
Supplement: Attachment — Submitted filename: Response to Reviewers.docx [file pone.0251385.s003.docx]

February 25, 2021

The Editor

PLOS ONE

Sir,

We hereby resubmit our earlier study for your consideration. We are grateful for and have attempted to address all the comments made by editors and reviewers. Find below a summary of our response:

ACADEMIC EDITOR#1: “consider to list figures as Fig 1, A,B,C etc”

*OUR REPLY: We have reduced the number of figures and modified the numbering to match the above format.*

ACADEMIC EDITOR#2: “the statistical analysis has to be better described in Materials and Methods”

*OUR REPLY: The statistical methods used were briefly described in Materials and Methods and the purpose for applying them stated. Lines 101-138.*

ACADEMIC EDITOR#3: “explain how they mentioned specificity and sensitivity of the various patterns without having calculated them based on ROC curves and AUC analysis”

*OUR REPLY: We corrected our use of the terms “specificity” and “sensitivity” and calculated and applied them as appropriate. We also introduced the AUC analysis of the ROC curve to compare the various models. Lines 115-136.*

ACADEMIC EDITORS#4: “we recommend that you deposit your laboratory protocols”

*OUR REPLY: We have deposited both of them in figshare.com and they have DOI:*

*1. Okafor O, Ezemba N, Onyishi N, Ezike K. S1_Table.csv. February 2021. doi:10.6084/m9.figshare.14067221.v1*

*2. Okafor O, Ezemba N, Onyishi N, Ezike K. S1_R_code. February 2021. doi:10.6084/m9.figshare.14068715.v1*

ACADEMIC EDITOR#5: “Please amend your current ethics statement to include the full name of the ethics committee/institutional review board(s) that approved your specific study.”

*OUR REPLY: We have done this in the Materials and Methods. Lines 88-93.*

ACADEMIC EDITOR#6: “ensure that you have discussed whether all data/samples were fully anonymized before you accessed them”

*OUR REPLY: We have included this detail in the Materials and Methods and in the online submission form. Lines 97-100.*

ACADEMIC EDITOR#7: “In the ethics statement in the manuscript and in the online submission form, please provide additional information about the patient records/samples used in your retrospective study, including: a) the date range (month and year) during which patients' medical records/samples were accessed.”

*OUR REPLY: We have included this information both in the manuscript and in the online submission form. Lines 88-90.*

ACADEMIC EDITOR#8: “In your Methods section, please provide additional information about the medical data/samples collected and the demographic details of the human subjects. Please ensure you have provided sufficient details to replicated the analyses such as a table of relevant demographic details.”

*OUR REPLY: A new table describing the demographic details of all the human subjects employed in the study has been inserted in the manuscript. Tables 1 and 2 in Results section.*

ACADEMIC EDITOR#9: “In your Methods section, please provide a description of the outcomes assessed in your study.”

*OUR REPLY: A detailed description of the clinical outcomes of the test patients has been provided in the manuscript. Lines 151-163.*

ACADEMIC EDITOR#10: “Please ensure you have discussed any potential limitations of your study in the Discussion, including study design, sample size and/or potential confounders.”

*OUR REPLY: We have included this in the Discussion section. Lines 441-445.*

ACADEMIC EDITOR#11: “please provide additional information regarding your statistical analyses.”

*OUR REPLY: All the statistical methods used were briefly described in the Materials and Methods. Lines 101-138. S1 R_code, our R working script, was deposited online at figshare.com (Available online at: doi:10.6084/m9.figshare.14102849.v1)*

ACADEMIC EDITOR#12: “Please include captions for your Supporting Information files at the end of your manuscript, and update any in-text citations to match accordingly.”

*OUR REPLY: This has been written out at the end of the manuscript; in-text citations have also been placed. Lines 550-559.*

REVIEWER-1#1: “I would suggest to the Authors to report in the Discussion section a brief view of the literature about the discrepancy between the clinical and histological diagnosis reported by different centers”

*OUR COMMENT: In the Discussion we have cited 5 earlier studies on this discrepancy and then extensively discussed the outcome and conclusions of one them (Walter AJ et al., Am J Obstet Gynecol. 2001), which indirectly influences our present study. Lines 345-361.*

REVIEWER-2#1: “Introduction, line 46, 47: please, cite some possible theory regarding etiology of TE”

*OUR COMMENT: We have cited 2 articles on this. Line 44.*

REVIEWER-2#2: “Introduction should be shorter”

*OUR COMMENT: The Introduction has been shortened from 638 words to 548 words.*

REVIEWER-2#3: “Material and methods, from line 106 to 114: this part should be moved in the results part”

*OUR COMMENT: These lines relating to the human subjects’ demographics and other related data have been moved to the Results section. Lines 140-164.*

REVIEWER-2#4: “Discussion: I would suggest discussing also when TE is impossible to be proved by histology (e.g.: diaphragmatic hernias). For instance, recently it has been reported (Viti A, et al. Endometriosis Involving the Diaphragm: A Patient-Tailored Minimally Invasive Surgical Treatment. World J Surg. 2020 Apr;44(4):1099-1104.) that up to 45% of patient with diaphragmatic endometriosis had no histological diagnosis. Please discuss on this.”

*OUR COMMENT: The emphasis of that study was on the surgical treatment of 21 cases of diaphragmatic endometriosis. In their study only 12 cases had samples analysed histologically, of which 10 were classified as endometriosis and 2 as "stromal-only" endometriosis. The other 9 cases were cases of diaphragmatic perforations in the absence of nodules and sample were not taken for histology i.e. not a case of incapacity to histologically detect endometriosis in samples sent. In those 9 cases the diagnosis of endometriosis therefore was based on symptomatology and/or prior history of confirmed abdominal/pelvic endometriosis. Our own study is based entirely on the histological diagnosis of endometriosis; and the surgical approach carried out in our hospital emphasises on thoroughly exploring the thoracic cavity and striving to obtain samples for histological analysis. As a consequence of this thoroughness there was no case of failure to establish a histological diagnosis in all the cases that had diaphragmatic lesions of any form. Thank you for giving me the opportunity to discuss this. We have not included this discussion in the manuscript because, as said earlier, our emphasis here is on making and improving the histological diagnostic yield on samples sent for analysis.*

REVIEWER-3#1: “First of all in endometriosis there are endometrioid glands and endometrioid stroma (not endometrial)”

*OUR COMMENT: We have made these changes.*

REVIEWER-3#2: “periods lines 63-68 are confusing; be concise; lines 68-70”

*OUR COMMENT: Thank you for pointing this ambiguity and imprecision out. We have attempted to clearly point out the divergent lines of thought that derive from an initial consideration, thus: “****In order to resolve this dilemma, the finding, on one hand, of long duration prior to diagnosis in spite of hospital presentation (possibly to different clinicians) will point to the clinicians as the cause of under-diagnosis. On the other hand, the correct and definitive diagnosis coming only after clinico-pathological reviews necessitated by non-resolution of symptoms, insistence of the clinicians or requests for second opinion will point to the histopathologist as the cause of the underdiagnosis and apparent rarity of the disease. The scenario is further compounded by a finding that less than 50% of laparoscopic biopsies of lesions visually diagnosed as endometriosis were confirmed to be endometriosis on histological study [8].****” Lines 58-67.*

REVIEWER-3#3: “delete lines 76-83; Insert the following:”

*OUR COMMENT: The deletions and insertions have been done. Lines 69-78.*

REVIEWER-3#4: “Material and methods: insert from results: The interval between the onset of symptoms 239 and definitive histopathological

diagnosis was 49 weeks (range = 1 week to 300 weeks).”

*OUR COMMENT: At the instance of the 2^nd^ Reviewer (REVIEWER-2#3:) we moved this phrase and other demographic aspects of the human subjects to Results section. Lines 144-164.*

REVIEWER-3#5: “There are too many figures. Delete figures 2, 4, and 11. Change the order of the rest:”

*OUR COMMENT: We deleted the 3 figures and changed the ordering and labels of the rest in accord with this comment. Thank you for the detailed commenting.*

REVIEWER-3#6: “Results: Change the order: Nine notable histological features ... Twenty-four cases (92%)... Three histological features ...”

*OUR REPLY: We have reordered the paragraphs in accord with this comment. Lines 165-209.*

REVIEWER-3#7: Structural modification of 4 paragraphs.

*OUR COMMENT: We have implemented all the essential changes of this structural reconstruction into the Discussion.*

Yours faithfully,

**DR. OKECHUKWU OKAFOR** *(DAPath, FMCPath, FICS)*
