# Supplementary material for: Improving the diagnostic recognition of thoracic endometriosis: Spotlight on a new histo-morphological indicator
Source: PLoS One. 2021 May 13;16(5):e0251385. doi: 10.1371/journal.pone.0251385 (PMC8118331; doi:10.1371/journal.pone.0251385)
Supplement: Attachment — Submitted filename: Response to Reviewers.docx [file pone.0251385.s004.docx]

April 19, 2021

The Editor

PLOS ONE

Sir,

We hereby resubmit our earlier study for your consideration. We are grateful for and have attempted to address all the comments made by editors and reviewers. Find below a summary of our response:

ACADEMIC EDITOR#1: “How were the various histopathological features identified?”

*OUR REPLY: We have described our methodology in a detailed manner in Lines 95-101 and referenced the supplementary table (S1 Table) that contains the results.*

ACADEMIC EDITOR#2: “For the statistical analysis, it is not necessary to explain the tests. The authors have to explain HOW they performed the tests.”

*OUR REPLY: The explanation of the statistical methods have been removed. In Lines 102-119 of Material and methods we fully described how the statistical analysis was performed and referenced the supplementary table (S2 Table) that contains the results.*

ACADEMIC EDITOR#3: “A R script cannot be presented as supplementary table. Again, data need to be understood by everyone. Please transform the R script in something that is understandable. Moreover, correct S1 R code all over the text.”

*OUR REPLY: We removed the R script as supplementary data and erased all reference to it in the article text.*

ACADEMIC EDITORS#4: “It is unclear how the cut offs for sensitivity and specificity were established. These explanations should be in the statistical analysis section.”

*OUR REPLY: We omitted the use of R code to obtain the best cutoff points but did it manually from the supplementary tables provided; we then described in detail how this was done in Lines 257-262 of the Results section.*

ACADEMIC EDITOR#5: “A great limit of the paper is that the reliability of the new diagnostic test should be evaluated in a different cohort. Although collecting a new cohort might be difficult, the Authors should declare this limit of the study in the Discussion section.”

*OUR REPLY: We have included this limitation in Lines 430-431 of Discussion section.*

REVIEWER-2: “diaphragmatic rupture might be the result of the involution of endometriosis tissue. What should the authors suggest to biopsy in these cases of no evidence of macroscopic endometriosis tissue? How did the authors manage similar cases? Maybe authors should also briefly describe in the results the macroscopical features of endometriosic lesions they resected.”

*OUR REPLY: We have briefly described the macroscopic features in Lines 149-151 of the Results section and have suggested what to biopsy in cases of no evidence of macroscopic endometriosis tissue in Lines 323-331 of Discussion section.*

Yours faithfully,

**DR. OKECHUKWU OKAFOR** *(DAPath, FMCPath, FICS)*
